# CiTO, the Citation Typing Ontology

**DOI:** 10.1186/2041-1480-1-S1-S6

**Published:** 2010-06-22

**Authors:** David Shotton

**Affiliations:** 1 Image Bioinformatics Research Group, Department of Zoology, University of Oxford South Parks Road, Oxford OX1 3PS, UK

## Abstract

CiTO, the Citation Typing Ontology, is an ontology for describing the nature of reference citations in scientific research articles and other scholarly works, both to other such publications and also to Web information resources, and for publishing these descriptions on the Semantic Web. Citation are described in terms of the factual and rhetorical relationships between citing publication and cited publication, the in-text and global citation frequencies of each cited work, and the nature of the cited work itself, including its publication and peer review status. This paper describes CiTO and illustrates its usefulness both for the annotation of bibliographic reference lists and for the visualization of citation networks. The latest version of CiTO, which this paper describes, is CiTO Version 1.6, published on 19 March 2010. CiTO is written in the Web Ontology Language OWL, uses the namespace http://purl.org/net/cito/, and is available from http://purl.org/net/cito/. This site uses content negotiation to deliver to the user an OWLDoc Web version of the ontology if accessed via a Web browser, or the OWL ontology itself if accessed from an ontology management tool such as Protégé 4 (http://protege.stanford.edu/). Collaborative work is currently under way to harmonize CiTO with other ontologies describing bibliographies and the rhetorical structure of scientific discourse.

## Structured digital abstract

Basic bibliographic, entity and project metadata relating to this article, recorded in a structured machine-readable form, is available as **Additional File **1 accompanying this paper, downloadable from http://dx.doi.org/10.1186/2041-1480-1-S1-S6/suppl/S1. This information is encoded as RDF (http://en.wikipedia.org/wiki/Resource_Description_Framework), serialized in Notation3 format (http://en.wikipedia.org/wiki/Notation3).

## Digital Object Identifier	

The DOI of this article is http://dx.doi.org/10.1186/2041-1480-1-S1-S6. 

## Background

### Context and rational

While the advent of on-line publishing and bibliographic search engines has made the problem of finding individual research articles considerably easier, the present scholarly citation system inadequately exposes the knowledge networks that exist within the scientific literature, linking papers, authors and research projects. Much of the problem stems from the lack of freely available citation data. In this Open Access age, it is a scandal that reference lists from journal articles, the core elements of the academic data cycle, are not freely available for use by scholars.

This paper describes CiTO, the Citation Typing Ontology, a new tool to permit the characterization of citations, and illustrates both how CiTO can be used to characterize citations, including the citations made within this paper, and how these data can be published in machine-readable form. If such CiTO-enabled machine-readable citation data were to be associated with all scholarly publications and published freely on the Web, the construction and interrogation of citation networks would become trivially simple, with enormous advantages to scholarship.

### What is CiTO

CiTO, the Citation Typing Ontology, is an ontology for describing the nature of reference citations in scientific research articles and other scholarly works, both to other such publications and also to Web information resources, and for publishing these descriptions on the Semantic Web. It has been designed with the requirements of biomedical researchers in mind. Citation are described in terms of the factual and rhetorical relationships between citing publication and cited publication, the in-text and global citation frequencies of each cited work, and the nature of the cited work itself, including its publication and peer review status. This paper describes CiTO and illustrates its usefulness both for the annotation of bibliographic reference lists and for the visualization of citation networks.

The latest version of CiTO, described in this paper, is CiTO Version 1.6, published on 26 March 2010. CiTO is written in the Web Ontology Language OWL, uses the namespace http://purl.org/net/cito/, and is available from http://purl.org/net/cito/. This site uses content negotiation to deliver to the user an OWLDoc Web version of the ontology if accessed via a Web browser, or the OWL ontology itself if accessed from an ontology management tool such as Protégé 4 [1], the ontology editor used in the construction of CiTO.

### What is meant by a citation

In the context of the Citation Typing Ontology, a bibliographic citation is a reference within a particular citing work to another publication (e.g. a journal article, a book chapter or a web page) termed the cited work. In scientific research articles, citations commonly take two forms: a condensed form within the text of the article (e.g. (Shotton and Attaran, 1998), or [14]), hereafter termed an *in-text citation*, and a full form within a reference list at the end of the article (e.g. Shotton, D.M. and Attaran, A. (1998). Variant antigenic peptide promotes cytotoxic T lymphocyte adhesion to target cells without cytotoxicity. *Proc. Natl. Acad. Sci. USA*. **95**:15571-15576.). This use of the word ‘citation’ should be clearly distinguished from the common related use of this word to indicate the cited work itself. Within CiTO, ‘cite’ and 'citation' denote the performative act of citation itself, not the target of that citation.

## CiTO scope and usage

### Citation publication and citation networks

The first purpose of CiTO is to enable the citations within a citing work to be recorded and published in machine-readable form as RDF [2], thus (serialized in Notation3 format [3]):

<http://example1.com/citingwork>**cito:cites**

<http://example2.com/citedwork> .

Even this simple statement that a citation exists opens significant possibilities, for example in enabling the easy creation of citation networks simply by combining the RDF citation lists from several papers.

Reciprocally we can say:

<http://example2.com/citedwork>** cito:isCitedBy**

 <http://example1.com/citingwork>  . 

which is useful in certain circumstances, despite the logical redundancy from a reasoning viewpoint.

**Figure 1:**
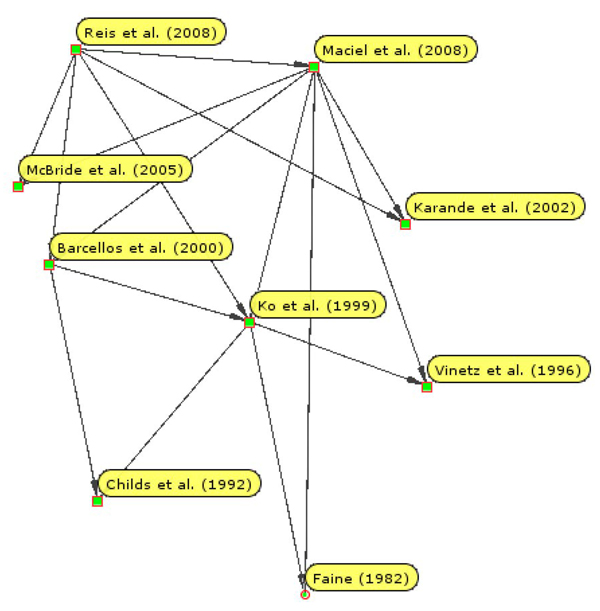
**A CiTO citation network** A citation network of selected articles directly or indirectly cited by Reis et al. (2008) [4], automatically displayed using the open source RDF graphing application Welkin, from an input RDF graph of *cito:cites* relationships.

Figure 1 shows a simple citation network linking a few papers directly or indirectly cited by Reis *et al*. (2008) [4], the target research article for our recent semantic publishing exemplar [5] described by Shotton *et al*. (2009) [6]. This diagram was created automatically by using an RDF graph of CiTO citations as input to the RDF graph visualization tool Welkin [7], with the nodes arranged along a vertical temporal axis.

The importance of having access to such citation network information in a readily computable form has recently been highlighted by the publication of an important paper by Greenberg [8], examining the biomedical literature. He constructed the complete citation network, comprising 242 papers and 675 citations, of all PubMed-indexed English-language papers between 1992 and 2007 addressing the very specific hypothesis that β amyloid, a protein accumulated in the brain in Alzheimer’s disease, is produced by and injures skeletal muscle of patients with inclusion body myositis. Importantly, he found

• unfounded authority being established by bias in citations, with papers that supported the hypothesis being cited in preference to papers that refuted or weakened it;

• amplification of the strength of the hypothesis in papers that presented no additional evidence in support; and

• the conversion of statements of the hypothesis in early papers into statements of 'fact' in later citing papers, through the act of citation alone.

Worryingly, he found these same trends present in applications for grants funded by the National Institutes of Health, obtained through the Freedom of Information Act.

Thus while citation is an impartial scholarly method and a powerful form of social communication, Greenberg was able, through analysis of this particular claim-specific citation network, to document distortions in its social use that included bias, amplification, invention of 'facts' and the creation of unfounded authority for claims.

All this shows how valuable analysis of citation networks can be, once you have the citation data are available. CiTO provides a way of encoding citation data in publishable machine-readable form, easing the task of creating of such citation networks.

### Citation characterization

The second purpose of CiTO is to permit characterization of bibliographic citations. The reasons that one publication cites others are varied. Usually, it is because the more recently published citing work has gained assistance of some sort, perhaps in the form of background information, ideas, methods or data, from the older cited works. It is for this reason that Google Scholar has as its strapline “Stand on the shoulders of giants”, echoing Sir Isaac Newton’s famous remark to his rival Robert Hooke "If I have seen a little further, it is by standing on the shoulders of Giants". However, more rarely, citations may be made to critique or refute previous works. CiTO makes it possible to capture and publish such distinctions, i.e. the intent of the author when citing a particular publication, permitting authors (or others) to create metadata describing their citations, quite distinct from metadata describing the cited works themselves. The full list of possible citation typing relationships presently recordable using CiTO is given in Table 1.

**Table 1:** The 23 relationships between citing and cited document in CiTO

Factual relationships	Rhetorical relationships		
	**Positive**	**Negative**	**Neutral**

*cito:cites*	*cito:confirms*	*cito:corrects*	*cito:discusses*
*cito:citesAsAuthority*	*cito:credits*	*cito:critiques*	*cito:reviews*
*cito:citesAsMetadataDocument*	*cito:extends*	*cito:disagreesWith*	
*cito:citesAsSourceDocument*	*cito:obtainsSupportFrom*	*cito:qualifies*	
*cito:citesForInformation*	*cito:supports*	*cito:refutes*	
*cito:isCitedBy*	*cito:updates*		
*cito:obtainsBackgroundFrom*			
*cito:sharesAuthorsWith*			
*cito:usesDataFrom*			
*cito:usesMethodIn*			

These relationships are all object properties within CiTO. With the exception of *cito:cites* and its inverse property *cito:isCitedBy*, all of the above are sub-properties of *cito:cites*. All the sub-properties of *cito:cites* always characterize the relationship *from* the citing work *to* the cited work, and their inverse properties are not employed. Thus *cito:supports* and *cito:obtainsSupportFrom* are separate and distinct properties, and are not the inverse of one another. A single citation can be characterized by several different relationships, both factual and rhetorical. It is for the user to decide which relationships are most appropriate, after consulting their ontology textual definitions (entered as 'Comments' in the data property annotation fields using Protégé 4). In Notation3 format, such characterizations can be made as follows:

 <http://example1.com/citingwork>

**cito:cites** <http://example2.com/citedwork> ;

**cito:usesMethodIn** <http://example2.com/citedwork> ;

**cito:extends** <http://example2.com/citedwork> ;

**cito:sharesAuthorsWith** <http://example2.com/citedwork> ; .

### Citation frequency

The third purpose of CiTO is to permit citation frequencies to be recorded, of two different types, *local* and *global*. We are familiar with journal impact factors, based on the global frequency of citation of the papers they contain by the scholarly community as a whole. Despite their vulnerability to abuse and 'spiking' [9,10], such impact factors are widely used to evaluate the quality of journals, and, less properly, as metrics for the quality of individual papers and the academic merits of their authors and institutions, on the crude premise that all citations are ‘votes of confidence’ in the cited papers. Another and lesser used aspect of citation frequency relates to the local importance of a cited publication to the citing publication. Put crudely, if Paper A cites Paper B once, but cites Paper C ten times at different points within the text, then, *from the point of view of the citing paper*, Paper C is more significant, irrespective of its global citation frequency relative to Paper B.

CiTO permits one to record both the in-text local citation frequency from Paper A to each of the papers it cites, and also the global citation frequency of each cited papers, as determined by consulting third-party authorities such as Google Scholar [11], the ISI Web of Knowledge [12] or SCOPUS [13] on a particular date. Such global citation counts providing proxy estimates of the importance of each cited paper to the whole academic community. In CiTO, such information is recorded using the following properties shown in Table 2. In-text and global citation information for particular cited publications can be recorded in the following manner.

<http://example1.com/citingwork>

**cito:cites** <http://example2.com/citedwork> ;

**cito:inTextCitationFrequency** [

  a **cito:InTextCitationCount** ;

**cito:inTextCountValue** "10"^^xsd:integer ;

**cito:inTextCitationTarget** <http://example2.com/citedwork> ;

 ] ; .

<http://example2.com/citedwork>

**cito:isCitedBy** <http://example1.com/citingwork> ;

**cito:globalCitationFrequency** [

  a **cito:GlobalCitationCount** ;

**cito:globalCountValue** "206"^^xsd:integer ;

**cito:globalCountSource** <http://scholar.google.com>;

**cito:globalCountDate** "2009-03-11"^^xsd:date** ;**

 ] ; .

**Table 2:** Entities used for citation frequency encoding in CiTO

Classes	Object properties	Datatype properties
*cito:GlobalCitationCount*	*cito:globalCitationFrequency*	*cito:globalCountDate*
*cito:InTextCitationCount*	*cito:globalCountSource*	*cito:globalCountValue*
	*cito:inTextCitationFrequency*	*cito:inTextCountValue*
	*cito:inTextCitationTarget*	

There is intentional redundancy in these sets of triples, since ‘A cites B’ and 'B is cited by A' could both be deduced from the other statements. This level of redundancy has a practical usefulness, since the direct citation statements can be used on their own to provide clean input to citation network visualization programs such as Welkin (Figure 1), and since the explicit reciprocal statement in the second set of triples would preserve the identity of the citing work if the 'citing' and 'cited' sets of triples were to be separated.

**Figure 2:**
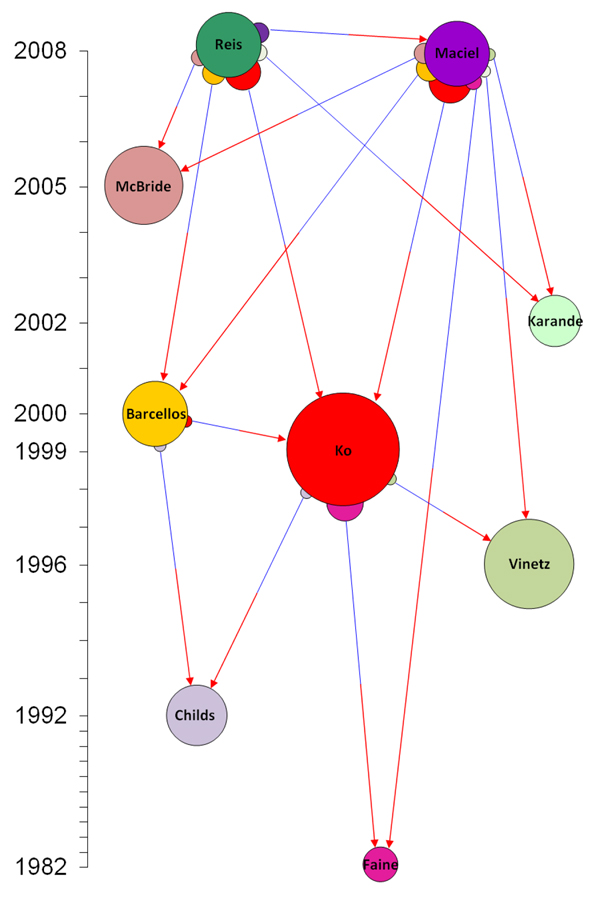
**An alternative representation of a CiTO citation network, displaying both in-text and global citation frequencies** This figure shows the same citation network as Figure 1, but was created manually to encode citation frequency information. The node size of each pre-2006 reference is proportional to the cube root of the number of global citations received, using numerical data from Google Scholar acquired on March 11 2009. The bumps on each citing reference are proportional to the square root of the number of in-text citations of the cited paper within each citing paper, an indication of the importance of the cited paper to the citing paper, and have a colour that matches the colour of the cited paper.

An alternative view of the citation network shown in Figure 1 is provided by Figure 2, in which the node size is proportional to the cube root of the number of global citations received by each pre-2006 reference, using numerical data from Google Scholar, while the bumps on each citing reference are proportional to the square root of the number of in-text citations of the cited paper within each citing paper, an indication of the importance of the cited paper to the citing paper.

### Characterization of cited works: use of the FRBR classification model

**Figure 3:**
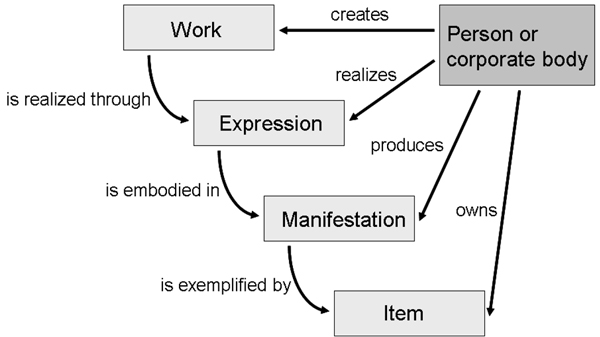
**The FRBR classification** The diagram shows the relationships between *Works*, *Expressions*, *Manifestations* and *Copies*, and their relationship to people and/or corporate bodies in the FRBR classification.

The fourth purpose of CiTO is to enable the cited works themselves to be characterized, so that someone reading a reference list marked up using CiTO can better appreciate their nature. In making this characterization, CiTO has adopted relevant aspects of the FRBR (Functional Requirements for Bibliographic Records) classification model [14] developed by the United States Library of Congress for characterizing different aspects of a publication (Figure 3). Readers may be interested to note that FRBR has recently been harmonized with the CIDOC Conceptual Reference Model (CIDOC CRM) [15,16] and represented as FRBRoo, an encoding of FRBR using the CIDOC CRM [17].

This FRBR classification distinguishes *Works*, *Expressions*, *Manifestations* and *Items*:

A *Work* is a distinct intellectual or artistic creation, an abstract concept recognised through its various expressions. An example of a *Work* is your latest research paper.

An *Expression* is the specific form that a *Work* takes each time it is ‘realized’ in physical or electronic form. For your latest research paper, Draft 5, the preprint, and the published version to which the publisher assigned a unique Digital Object Identifier (DOI) [18], are all *Expressions* of the same work.

A *Manifestation* of an expression of a scholarly work defines its particular physical or electronic embodiment. If your latest research paper appeared as an article in a print journal, in the on-line version of that journal as an HTML page, and also as a downloadable PDF file, these are three separate manifestations of the same ‘version of record’ *Expression* of your work, all bearing the same DOI, which can be viewed as alternate 'containers' or 'channels' for the same information..

In FRBR, an *Item* is one single exemplar copy of a *Manifestation*, i.e. a physical or electronic object that can be owned by a person, for example a printed copy of a journal article on your desk, or a PDF file of that article that you purchased from a publisher and that now resides in digital form on your computer hard drive.

In CiTO, the definition of *cito:Work* is restricted to works that cite or are cited, primarily works of scholarship that contain bibliographic references, and excludes artistic works such as plays or photographs that do not. Additionally, while the original FRBR specification is rather vague as to whether the FRBF classification applies to digital as well as physical *Manifestations* of *Expressions*, CiTO certainly does. For these reasons *cito:Work* is a subclass of *frbr:Work*, not an equivalent class.

However, since *cito:Work* covers both citing and cited works, and since certain things that are cited in academic papers might not themselves be strictly considered as works of scholarship (e.g. blog entries, newspaper articles, and the web sites of the suppliers of scientific reagents and equipment), the term "Work" is employed in CiTO, rather than the more restrictive term "ScholarlyWork".

As a logical consequence of *cito:Work* being a subclass of *frbr:Work*, *cito:Expression* and *cito:Manifestation* are also subclasses of their respective FRBR classes. Since normal bibliographic citations are not made to Items, CiTO does not include this class.

**Figure 4:**
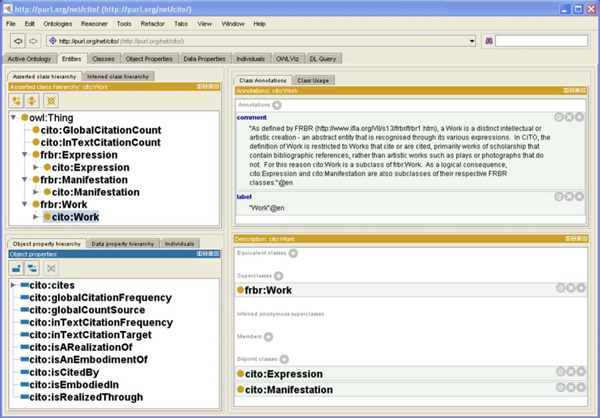
**The CiTO Ontology** A screenshot of the ontology editor Protégé 4, showing the upper-level classes and object properties of CiTO, and the relationship of these classes with the corresponding FRBR classes.

These upper-level classes of CiTO are shown in Figure 4. *cito:Work* is related to *cito:Expression* by the object property *isRealizedThrough*, with the inverse property *isRealizationOf*, while *cito:Expression* is related to *cito:Manifestation* by the object property *isEmbodiedIn*, with the inverse property *isEmbodimentOf*.

On first encounter, the FRBR classification into Works, Expressions, Manifestations and Items might seem a little fussy, and its application to CiTO, detailed below, appears occasionally to result in apparently redundant terminology, e.g. Work: *cito:Report*; Expression: *cito:ReportDocument*. However, this level of granularity of description is of enormous value, since it avoids ambiguities of meaning that abound in 'flatter' bibliographic ontologies, as discussed below.

CiTO defines 41 disjoint subclasses of *cito:Work* and 43 disjoint subclasses of *cito:Expression,* shown in Tables 3 and 4 respectively, to suit typical biomedical scientific usage.

**Table 3:** The 41 sub-classes of *cito:Work*

Sub-classes of *cito:Work*	Sub-sub-classes of *cito:Work*
*cito:Biography*	
*cito:CaseForSupport*	
*cito:Dataset *	
*cito:GrantApplication*	
*cito:Image*	
	*cito:MovingImage*
	*cito:StillImage*
*cito:InstructionalWork*	
*cito:Metadata*	
	*cito:BibliographicMetadata*
	*cito:CitationMetadata*
	*cito:EntityMetadata*
	*cito:ProjectMetadata*
*cito:Model*	
*cito:NewsItem*	
*cito:Opinion*	
*cito:Proposition*	
*cito:ReferenceWork*	
*cito:Report*	
	*cito:ClinicalCaseReport*
	*cito:ClinicalTrialReport*
*cito:ResearchPaper*	
*cito:Review*	
	*cito:BookReview*
*cito:Specification*	
	*cito:Algorithm*
	*cito:AuthorityFile*
	*cito:Catalog*
	*cito:ClincialTrialDesign*
	*cito:ExperimentalProtocol*
	*cito:MinimalInformationStandard*
	*cito:Ontology*
	*cito:Patent*
	*cito:PatentApplication*
	*cito:ReportingStandard*
	*cito:StandardOperatingProcedure*
	*cito:Taxonomy*
	*cito:TechnicalStandard*
	*cito:Workflow*
*cito:Thesis*	
*cito:WorkingPaper*	

**Table 4:** The 45 sub-classes of *cito:Expression*

Sub-classes of *cito:Expression*	Sub-sub-classes of *cito:Expression*	Sub-sub-sub-classes of *cito:Expression*
*cito:Database*		
*cito:Document*		
	*cito:Abstract*	
	*cito:Book*	
	*cito:BookSection*	
		*cito:BookChapter*
	*cito:CaseForSupportDocument*	
	*cito:ConferencePaper*	
	*cito:ConferencePoster*	
	*cito:ConferenceProceedings*	
	*cito:Email*	
	*cito:GrantApplicationDocument*	
	*cito:Letter*	
	*cito:Manuscript*	
	*cito:PatentApplicationDocument*	
	*cito:PatentDocument*	
	*cito:PeriodicalIssue*	
		*cito:JournalIssue*
		*cito:MagazineIssue*
		*cito:NewspaperIssue*
	*cito:PeriodicalItem*	
		*cito:Editorial*
		*cito:JournalItem*
		cito:JournalArticle*
		*cito:MagazineArticle*
		*cito:NewspaperArticle*
	*cito:Preprint*	
	*cito:PressRelease*	
	*cito:ReportDocument*	
	*cito:SupplementaryInformation*	
*cito:Figure*		
*cito:Periodical*		
	*cito:Journal*	
	*cito:Magazine*	
	*cito:Newspaper*	
*cito:PersonalCommunication*		
*cito:Presentation*		
*cito:Software*		
*cito:Spreadsheet*		
*cito:Table*		
*cito:WebContent*		
	*cito:BlogEntry*	
	*cito:WikiEntry*	

* *cito:JournalArticle* is a sub-class of *cito:JournalItem*.

Although in current scholarly practice citations do not usually required specification of the Manifestation of the cited Expression of a Work, CiTO does for convenience permit the nature of the Manifestation to be recorded in terms of the 5 disjoint subclasses of *cito:Manifestation* shown in Table 5. 

**Table 5:** The 5 sub-classes of *cito:Manifestation*

Sub-classes of *cito:Manifestation*	Sub-sub-classes of *cito:Manifestation*
*cito:ComputerFile*	
	*cito:DigitalMediaFile*
	*cito:OnlineFile*
*cito:PrintObject*	
*cito:WebPage*	

Clearly, these subclasses of *cito:Work*, *cito:Expression* and *cito:Manifestation* are not exhaustive. They are not meant to be. The purpose of CiTO is to be as simple as possible while yet being fit for purpose to characterize biomedical citations in the new digital world. Cited works are more completely described in other ontologies, as discussed below.

### To what do citations refer?

At the fundamental philosophical level, the target of a citation is the Work itself, rather than any particular Expression or Manifestation of that Work. However, there are three pragmatic reasons why the object of a CiTO citation should normally be an Expression of a particular Work.

First, publication of RDF citation information as Open Linked Data requires that both the citing work and the cited work are referenced by means of Uniform Resource Identifiers (URIs). Works in FRBR are abstract concepts, and as such are typically not assigned URIs It is only the published 'version of record' of a paper that is assigned a DOI, which can be used to create such a unique dereferenceable URI. (Any DOI (e.g. doi:10.1371/journal.pntd.0000228.x001) may be turned into a resolvable URI by substituting "http://dx.doi.org/" for the initial "doi:" and using this as the address in a Web browser, employing the International DOI Foundation's automatic DOI resolution service to obtain the true URI for the paper (in this case http://purl.org/net/semanticpublication/pntd.0000228). Alternatively, for *cito:WebContent*, it is the Manifestations as *cito:WebPages* that have citable URIs.

Second, while in principle the citation holds true for any Expression of the Work, for example a translation into another language, in reality the object of the citation originally made by the author on a particular day was a particular Expression of the Work, namely a particular published ‘version of record’ that he or she first located, then read and finally cited.

Thirdly, CiTO may be used to specify the number of in-text citations to the cited Work, and the number of global citations that the cited work has received at the time of local citation. The number of in-text citations to a particular cited Work within your most recent research paper, and also the total number of distinct references cited, probably changed as the paper was developed through various drafts. Thus the version that matters for CiTO in determining the number of in-text citations is the final published ‘version of record’ Expression of your own published paper. Similarly, the version of the cited article that matters for determining the global citation counts is its 'version of record' Expression, since it is only that of which citing third parties are normally aware and to which their citations are directed.

For these reasons, the domain and range of *cito:cites* are constrained to *cito:Work*, *cito:Expression* or *cito:Manifestation*.

If an author wishes, when using CiTO, to add citation typings to references cited within his or her own citing work *prior* to publication, the blank node *_:ThisWork* may be employed to denote the author's citing work. This can subsequently be replaced by the URI of the unique DOI of the ‘version of record’ when the author's citing paper is published.

### Other CiTO capabilities

The publication status and the peer-review status of an expression of a work can also optionally be recorded:

**Peer review status**: *cito:peerReviewed*, a Boolean data property having the value *True* if the cited work has been peer reviewed, or *False* if the cited work has not been peer reviewed.

Such information could, for example, enable searches designed to retrieve only peer-reviewed articles.

**Publication status**: *cito:unpublished*, a Boolean data property having the value *True* if the cited work has not been published, or *False* if the cited work has been published.

Such characterization could be used to refer to a preprint in an open access institutional repository of a paper yet to appear as a published 'version of record' journal article with an assigned DOI.

CiTO thus has a number of subclasses of *cito:Work*, *cito:Expression* and *cito:Manifestation* that enable accurate characterization of cited publications. When using CiTO for this purpose, publications should be characterized using a single subclass of *cito:Work* and a single subclass of *cito:Expression*. Each *cito:Expression* can optionally also be given a *cito:Manifestations* type, a publication status and a peer review status, as in the following example:

<http://example2.com/citedwork>

 dcterms:bibliographicCitation "Full bibliographic details" ;

 rdfs:label "FirstAuthor et al. (Year)"; # label

 cito:isRealizationOf **cito:ResearchPaper** ; # work type

 rdf:type **cito:JournalArticle** ; # expression type

 cito:isEmbodiedIn **cito:WebPage** ; # manifestation type

**cito:unpublished** "false"^^xsd:boolean ; # publication status

**cito:peerReviewed** "true"^^xsd:boolean ; # peer review status

### CiTO vocabulary definitions

Great effort has been made during the creation of CiTO to give full and informative definitions to all its classes and properties. These definitions are given in the ontology itself [19] and in textual form in the **Additional File **2 accompanying this paper [20].

CiTO adopts the Dublin Core Metadata Initiative (DCMI) Type Vocabulary [21] definitions for the terms *cito:Dataset*, *cito:Image*, *cito:MovingImage*, *cito:Software,* and *cito:StillImage*. Other CiTO class names include all items in the vocabulary defined by SWAP (see below) for subclasses of the dc:type property *Text*.

CiTO extends the vocabularies mentioned above by defining relationships between citing and cited works, and by including a number of additional sub-classes of *cito:Work*,* cito:Expression* and* cito:Manifestation*, which have been created with the specific needs of the biomedical research community in mind.

Any possible future expansion of CiTO to fulfil the citation needs of other disciplines will require engagement with appropriate community domain experts. For example, classical scholarship in the commentary tradition requires comparison of textual variations between individual manuscripts (using the traditional meaning of the word, i.e. unique hand-written documents). Here, the FRBR concept of Item becomes important, but, for these unique creations, the distinctions between Expression and Manifestation, and between Manifestation and Item becomes blurred.

### Granularity and scope

The commentary tradition of classical and biblical scholarship has well-developed methods for citing individual sections, paragraphs or verses of cited works. In contrast, modern scientific citations are typically made to the cited works as complete entities. It was to enhance this standard practice that CiTO was developed. However, there are currently calls to permit a scientific article to be created compositionally from a set of pre-defined independent parts [22-24], and for individual rhetorical elements within the text to be referenced directly [25,26]. Indeed, it is perfectly possible, using hidden XML or RDFa code behind the displayed human-readable Web document, for the text of an on-line article to be marked up semantically to the level of the paragraph, the sentence or even the individual word, or to particular rhetorical elements (hypotheses, claims, supporting statements, refutations, etc.). Various tools to enable that to be done are in early-stage development, and such moves will require support from appropriate ontologies.

## The relationship of CiTO with other metadata schemas and ontologies

### CiTO and FRBR

The relationship of CiTO to FRBR has already been discussed. The FRBR classification of Work, Expression and Manifestation is fundamental to the structure of CiTO.

### CiTO and SWAP

The Scholarly Works Application Profile (SWAP) [27] describes the metadata requirements for a scholarly work. SWAP, like CiTO, follows the FRBR model, but its scope is different from that of CiTO, in that SWAP concerns itself with items of metadata surrounding the scholarly work that fall outside the scope of a bibliographic citation, such as funding agency and copyright holder. Conversely, CiTO is concerned with the factual and rhetorical relationships *between* citing and cited works, something which cannot be captured within the metadata of a single work. As far as possible, CiTO has adopted SWAP's terminology and class definitions. Unfortunately, SWAP lacks an accompanying RDF schema.

### CiTO and BIBO

Among many previous efforts to create metadata schemas and ontologies for characterizing bibliographic references, BIBO, the Bibliographic Ontology [28] written in OWL, provides the much-needed ability to describe the nature of cited works in RDF to a high degree of granularity, in terms of Title, Abstract, Journal, Volume, Pages, ISSN, DOI, dataCopyrighted, editor, etc. In addition to covering conventional scholarly works, BIBO also covers things outside that realm, including time lines, broadcasts (e.g. Interviewer, Performer, Producer) and legal entities (e.g. CourtReporter, Hearing, LegalCaseDocument). However, it is lacking equivalent classes for the majority of the CiTO subclasses of *cito:Work*, *cito:Expression* and *cito:Manifestation*, e.g. *cito:JournalArticle*, *cito:SupplementaryInformation* and *cito:ConferencePaper*, terms that are of central importance in academic citations.

Unfortunately, BIBO has not adopted the Work, Expression, Manifestation classification of FRBR, which leads to lack of precision in its nomenclature. For example, while CiTO has Work: *cito:ResearchPaper*; Expression: *cito:JournalArticle*, BIBO has *bibo:AcademicArticle*, which conflated these two concepts, and does not permit descriptions of alternative forms of Expression of the research paper, e.g. *cito:BookChapter*. While BIBO has *bibo:BookSection*, there is no way of specifying that such a book section is an alternative Expression of a research paper. Similarly, BIBO's definition of *bibo:Standard* is "A document describing a standard", whereas CiTO has Work:* cito:TechnicalStandard* ("A defined specification or requirement for a technical method, practice, process or protocol involved in, for example, manufacturing, computation, electronic communication, or digital media."); and Expression: *cito:Document* (A physical or electronic Expression of a Work, conveying a body of information primarily in textual form).

The only relationships in BIBO of potential relevance for the characterization of citations themselves are *bibo:affirmedBy*, *bibo:annotates*, *bibo:reviewOf* and *bibo:translationOf*. While CiTO also the related terms *cito:reviews* and* cito:supports*, the other two terms are unique and useful.

### CiTO and SWAN

SWAN (Semantic Web Applications in Neuromedicine) [29] is a project to develop knowledge bases for the neurodegenerative disease research communities. Within a set of modular ontologies created within SWAN [30] is the SWAN Scientific Discourse Relationships Ontology [31], designed for characterization of rhetorical statements within text. 

The purpose of the SWAN Scientific Discourse Relationships Ontology is to characterize the rhetorical structures that exist within scientific writings. For example, it can be used to encode the related triples *Statement_A derivedFrom JournalArticle* and *Statement_A refersTo GeneX*. Its primary purpose is therefore wider than that of CiTO. Nevertheless, the SWAN Scientific Discourse Relationships Ontology includes the following relationship terms identical or similar to those for relationships within CiTO, although the targets of and the definitions for those relationships are subtly different.

### Future revisions of CiTO, and harmonization with other ontologies

An active collaboration is ongoing, as an activity of the Scientific Discourse Task Force of the W3C Semantic Web for Health Care and Life Sciences Interest Group [32] that includes this author, those involved in the SWAN project, and other interested parties, to harmonize CiTO and the SWAN Scientific Discourse Relationships Ontology, and to distinguish more clearly the role of CiTO in describing citations (including support for citation counts, citation characterization and citation networks) from that of the SWAN Scientific Discourse Relationships Ontology in describing the wider rhetorical structures that exist in scientific writings.

During future work, CiTO will also be more fully integrated with other vocabularies, for example by relating *cito:cites* with *dc:references*. The outcomes of these collaborations and revisions to CiTO, which are anticipated to result in the publication of CiTO Version 2.0, will be reported in due course.

Since the primary purpose of CiTO is to characterise citations, while that of BIBO is to characterize cited works, these two ontologies are essentially orthogonal. Subsequent work is anticipated that will harmonize CiTO with BIBO as much as is possible, given their differing fundamental structures.

## Examples of CiTO in use

**Figure 5:**
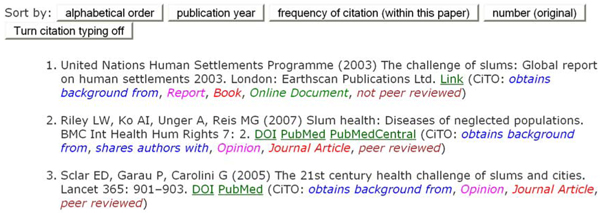
**An example of the use of CiTO to annotate a reference list** The first three references from the reference list of the enhance version of Reis et al. (2008) [4], with the citation typing display turned on. Above the references are buttons to re-order the references, and to turn off the citation typing display. This figure was first published in Shotton *et al*. [6].

The first example of the use of CiTO for annotation of the reference list in an on-line biomedical research article can be seen in our enhanced version of Reis *et al*. (2008) [5], which used CiTO v1.3. Here, the human readable CiTO mark-up can be made visible by first going to the References section of the paper (click the ‘References’ tab above the article’s title), and then by turning on the optional citation typing display (click the ‘Turn citation typing on’ button just before the first reference). Figure 5 provides a snapshot of the CiTO mark-up of the first few references in that paper. In this, the CiTO terms used, shown in colour and in italics, have been rendered more readable than the class or property labels in the ontology itself, by reverting from single-word class names in CamelCase (e.g. *cito:obtainsBackgroundFrom, cito:JournalArticle*) to normal English (i.e. *obtains background from*, *Journal Article*). An exemplar downloadable file containing all the references from that article with their CiTO mark-up and their citation frequency information in RDF N3 format is also available [33].

In a similar manner, CiTO v1.6 has been used to annotate the citations within the following reference list of this article. These annotated references are also available in a structured machine-readable form in the **Additional File **3 accompanying this paper [34].

## Conclusion

The first public version of CiTO, CiTO Version 1.3, was published on 5 May 2009 and was described in a preliminary report [35]. CiTO version 1.4, published on 24 November 2009, represented the first major extension of the ontology. A further revision, CiTO version 1.5, involved addition of FRBR classes and further CiTO classes and properties, was published on the Web on 1 February 2010. The current version, CiTO version 1.6, represents a significant further revision of the ontology, removing some unnecessary classes, simplifying the logical structure, and ensuring that all sibling classes and properties are disjoint. Relative to CiTO Version 1.3, the current version has 3 new FRBR classes added, and has 9 new object properties, 1 new data property, 20 new, 3 renamed and 5 deprecated subclasses of *cito:Work*, 26 new, 1 renamed and 2 deprecated subclasses of *cito:Expression*, and 1 new and 3 renamed subclasses of *cito:Manifestation*, giving a total of 98 classes, 31 object properties and 5 data properties in the current version. Full details of the differences between these versions are given in Additional File 2  [20].

The reported extensions and revisions that have led to the current Version 1.6 of CiTO are possible at this early stage of the ontology's life, since our own published metadata files using CiTO v1.3 have been updated, and since CiTO has not yet been widely used elsewhere.

In developing CiTO, I have sought to create an ontology sufficient in scope for the types of bibliographic citation encountered in biomedical research articles. Authors should be able to use it to type their own citations, although there is clearly scope for the development of an ontology-backed tool (e.g. a Word plug-in) that would assist authors in that process during paper writing. Alternatively, citation typing can be made at the time of publication or later.

CiTO is published as open source under a Creative Commons attribution license, and I invite engagement from interested members of the community in its use and extension to serve other domains, and in the development of authoring tools that can use it.

## Competing interests

The author declares that he has no competing interests.

## Supplementary Material

Additional File 1 Format: Text file in RDF Notation3 Format.
DOI: http://dx.doi.org/10.1186/2041-1480-1-S1-S6/suppl/S1.
Additional File 1 accompanying this paper  [36] contains metadata describing this article, recorded in a structured machine-readable form, encoded as RDF, and serialized in Notation3 format. Click here for file

Additional File 2 Format: HTML.
DOI: http://dx.doi.org/10.1186/2041-1480-1-S1-S6/suppl/S2.
Additional File 2 accompanying this paper [20] contains detailed description of CiTO version 1.6, including the definitions of each class and property, and a record of its differences from CiTO version 1.3. This file is published as a human-readable Web document in HTML format. Click here for file

Additional File 3 Format: Text file in RDF Notation3 Format.
DOI: http://dx.doi.org/10.1186/2041-1480-1-S1-S6/suppl/S3.
Additional File 3 accompanying this paper [34] contains the twelve bibliographic references and the twenty nine Web sites references, typed using the Citation Typing Ontology. The information is recorded in a structured machine-readable form, encoded as RDF^2^, serialized in Notation3 format^3^.
**Additional Files 1 and 3** complement one another, and together provide full metadata for this paper and its references. They are presented as an example of the metadata that should be freely published for all articles. Click here for file
