# Supplementary material for: CiTO, the Citation Typing Ontology
Source: J Biomed Semantics. 2010 Jun 22;1(Suppl 1):S6. doi: 10.1186/2041-1480-1-S1-S6 (PMC2903725; doi:10.1186/2041-1480-1-S1-S6)
Supplement: Additional File 2 — Format: HTML. DOI: http://dx.doi.org/10.1186/2041-1480-1-S1-S6/suppl/S2. Additional File 2 accompanying this paper [20] contains detailed description of CiTO version 1.6, including the definitions of each class and property, and a record of its differences from CiTO version 1.3. This file is published as a human-readable Web document in HTML format. [file 2041-1480-1-S1-S6-S2.htm]

CiTO, the Citation Typing Ontology


**CiTO, the Citation Typing
Ontology**

David Shotton (2010) *Journal of Biomedical Semantics* 1
(Suppl 1): S6.

**Additional
File 2:  The CiTO Vocabulary and Definitions**

**Self-citation, provenance,
copyright and license information**

The URL of this document is http://dx.doi.org/10.1186/2041-1480-1-S1-S6/suppl/S2.

This
document was written by David Shotton, Image Bioinformatics Research Group,
Department of Zoology, University of Oxford (david.shotton@zoo.ox.ac.uk), and
was last modified on 15 March 2010. 

©
2010 David Shotton.  This document is an open access reference work
distributed under the terms of the Creative Commons Attribution License 2.5 (http://creativecommons.org/licenses/by/2.5/),
which permits unrestricted use, distribution, and reproduction in any medium,
provided that the original author and source are attributed.

**History**

The
version of CiTO that this document describes is **CiTO Version 1.6**,
published on 26 March 2010.  CiTO is written in the Web Ontology Language
OWL, uses the namespace http://purl.org/net/cito/, and is available from http://purl.org/net/cito/.  Version
1.3 of CiTO, the first public version, was published on 5 May 2009.  The
ontology went through two further intermediate versions, Version 1.4 published
on 24 November 2009. and Version 1.5 published on 1 February 2010, documentation
of which are not separately published.  Changes between Versions 1.3 and
1.6 are detailed at the end of this document.

**Introduction**

CiTO,
the Citation Typing Ontology, is an ontology
for describing the nature of reference citations in scientific research
articles and other scholarly works, both to other such publications and also to
Web information resources, and for publishing these descriptions on the
Semantic Web.  This version of CiTO, CiTO Version 1.6, is described in the
following paper for which this is Supplementary File S2:

Shotton, D (2010).  CiTO, the Citation Typing
Ontology.  *J. Biomedical Semantics* 1 (Suppl 1): S6, doi:10.1186/2041-1480-1-S1-S6.

This
supplementary file describes the CiTO Vocabulary in greater detail, giving a textual
definition for each term in the annotation 'Comment' field. The terms names
given below are the *labels* used for the object properties and classes in the Citation Typing
Ontology, which are identical to the class and property names.  The
definitions given in this document are identical to the textual definitions in
CiTO itself. 

**Namespace**

The
namespace for CiTO is http://purl.org/net/cito/.  The URI of each term in
the ontology (e.g. *cito:Book*) is thus formed by using the term name
preceded by 'http://purl.org/net/cito/' (e.g. http://purl.org/net/cito/Book).

**Colours**

The
colours used for properties and classes in this document are the same as those
employed in the first on-line example of CiTO’s use given at http://dx.doi.org/10.1371/journal.pntd.0000228.x001#refs,
which can be revealed by clicking on the “Turn Citation Typing On” button at
the start of the References section. These colours, which are employed for
convenience only, and have no semantic meaning other than to distinguish CiTO
terms of different types, are:

Object Properties characterizing citations                                                                                          **blue**

*Work* and it
sub-classes
                                         
                                                             **pink**

*Expression*
and its sub-classes
                               
                                                           **red**

*Manifestation*
and its sub-classes
                          
                                                          **green**

*Data Property*
relevant to the Publication Status or Peer Review
Status                       
  **brown**

*Classes*, *Object
Properties* and *Data Properties* relevant to Citation Frequencies                    **orange**

Object Properties relating *Work*, *Expression*
and *Manifestation***black**

**CiTO Object Properties:**

Most
of these relationships describe why a citing Work cites a cited Work. When
using CiTO, a single citation should be described as having one or more of
these relationships, which are both factual and rhetorical in nature.

*cito:cites*  A statement of fact that the citing Work cites
the cited Work. [This is implicit in human-readable reference lists, and need
not be stated when annotating them using CiTO.  However, the statement is
useful in machine-readable metadata for construction of citation networks and other
purposes.] [For scholarly Works, it is usually the final published Expression
of the Work, known to publishers as the 'version of record', which is critical
for CiTO, since it is the references within this version of record that are
definitive, and it is the version of record itself that is cited by
others.  However, for certain items such as a Database or a BlogEntry, it
is the on-line Manifestation, i.e. the Web page URI, that is cited.  For
this reason, the range of cito:cites is left unconstrained.  (Inverse property: *cito:**isCitedBy*.)

*cito:isCitedBy*A statement of fact that the cited
Work is cited by the citing Work. (Inverse property: *cito:**cites*.)

There are 21 subclasses of *cito:**cites*, having the relationship
*from* the citing Work A *to* the cited Work B (direction always A to
B):

*cito:citesAsAuthority*The citing Work refers to the cited
Work as providing an authoritative description or definition of the subject
under discussion.

*cito:citesAsMetadataDocument*The citing Work refers to the cited
Work as containing metadata about the citing Work. (Inverse property: *cito:citesAsSourceDocument*.)

*cito:citesAsSourceDocument*The citing Work refers to the cited
Work as the source about which the citing Work contains metadata. (Inverse
property: *cito:citesAsMetadataDocument*.)

*cito:citesForInformation*The citing Work refers the reader to
the cited Work as a source of information on the subject discussed in the text.

*cito:confirms*The citing Work confirms facts, ideas
or statements presented in the cited Work.

*cito:corrects*  The citing Work corrects ideas or statements
presented in the cited Work.

*cito:credits*  The citing Work acknowledges contributions made
by the cited Work.

*cito:critiques*The citing Work critiques facts,
ideas or statements presented in the cited Work.

*cito:disagreesWith*The citing Work disagrees with ideas
or statements presented in the cited Work.

*cito:discusses*The citing Work discusses facts,
ideas or statements presented in the cited Work.

*cito:extends*  The citing Work extends facts, ideas or
understandings presented in the cited Work.

*cito:obtainsBackgroundFrom*The citing Work obtains background
information from the cited Work.

*cito:obtainsSupportFrom*The citing Work obtains intellectual
or factual support from the cited Work.

*cito:qualifies*The citing Work qualifies or places
conditions upon facts, ideas or statements presented in the cited Work.

*cito:refutes*  The citing Work refutes ideas or statements
presented in the cited Work.

*cito:reviews*The citing Work reviews ideas or statements
presented in the cited Work.

*cito:sharesAuthorsWith*The citing Work has at least one
author in common with the cited Work.

*cito:supports*The citing Work provides intellectual
or factual support for the cited Work*.*

*cito:updates*  The citing Work updates facts, ideas or
understandings presented in the cited Work.

*cito:usesDataFrom*  The citing Work used data from the cited Work.

*cito:usesMethodIn*The citing Work uses a method
described in the cited Work.

**CiTO Work, specifying the
nature or type of a citing or cited Work, and its
sub-classes**

*cito:Work*As defined by FRBR (http://www.ifla.org/VII/s13/frbr/frbr1.htm),
a Work is a distinct intellectual or artistic creation - an abstract entity
that is recognised through its various Expressions.  In CiTO, the
definition of Work is restricted to Works that cite or are cited, primarily
works of scholarship that contain bibliographic references, and excludes
artistic works such as plays or photographs that do not.  For this reason *cito:Work*
a subclass of *frbr:Work*.  Consequently, *cito:Expression* and *cito:Manifestation*
are also made subclasses of their respective FRBR classes.

When using CiTO, a Work
should be assigned to one of the following  **41 disjoint sub-classes**:

*cito:Algorithm*A precise sequential set of
pre-defined logical rules or computational operations to be employed for
solving a particular problem in a finite number of steps.(Subclass
of *cito:Specification*.)

*cito:AuthorityFile*A controlled vocabulary or official
list that establishes, for consistency, the authoritative forms of headings,
preferred terms or proper names to be used when creating a catalogue, or when
indexing and searching a set of entities within a defined domain. 
(Subclass of *cito:Specification.*)

*cito:BiographicMetadata*Standard bibliographic metadata
describing an Expression of a Work, including, to take the example of a Journal
Article, the author(s), the date of publication, the title, the journal name
and volume number, the first and last page numbers, and the Digital Object
Identifier.  (Subclass of *cito:Metadata.*)

*cito:Biography*An account of the events, works and
achievements, personal and professional, during the life of a person, either
living or dead.

*cito:BookReview*A written Work consisting of a review
and critical analysis of the content, scope and quality of a book or other
monographic work.  (Subclass of *cito:Review*.)

*cito:CaseForSupport*An accompaniment to a Grant
Application that provides a description of a proposed project and gives reasons
why it is worthy of funding. (See also *cito:GrantApplication.*)

*cito:Catalog*A
list of items describing the content of a resource, for example items in an
exhibition, offered for sale by a vendor, or contained within a library or
collection.  Ideally, Catalogs are created according to specific and
uniform principles of construction and are under the control of an Authority
File.  (Subclass of *cito:Specification.*)

*cito:CitationMetadata*Metadata describing the citations
made within an Expression of a Work to Expressions of other Works, and
(optionally) some characteristics of the cited Expressions.  (Subclass of*cito:Metadata.*)

*cito:ClinicalCaseReport*A presentation of findings following
a clinical or medical investigation on a human or animal patient, that may
contain a diagnosis and proposals for therapeutic treatment and/or
epidemiological control measures, or may propose further evaluative studies
that will eventually lead to such outcomes.  (Subclass of *cito:Report.*)

*cito:ClinicalTrialDesign*  A predefined written procedural method,
designed to ensure reliability of findings, for undertaking a medical or
veterinary clinical study of the safety, efficacy, or optimum dosage schedule
of one or more diagnostic, therapeutic or prophylactic drugs or treatments, or
of devices or techniques, involving a randomized controlled trial for
evidence-based assessment in humans or animals, specifying criteria of
eligibility, nature of controls, sampling schedules, data collection
parameters, statistical analyses, reporting standards, etc. to be employed in
undertaking the clinical trial.  (Subclass of *cito:Specification.*)

*cito:ClinicalTrialReport*The report of a pre-planned medical
or veterinary clinical study of the safety, efficacy, or optimum dosage
schedule of one or more diagnostic, therapeutic or prophylactic drugs, or of
devices, treatments or techniques, involving a randomized controlled trial for
evidence-based assessment in humans or animals selected according to
predetermined criteria of eligibility and observed for evidence of favourable
and unfavourable effects.  (Subclass of *cito:Report.*)

*cito:Dataset*A collection of related facts, often
expressed in numerical form and encoded in a defined structure.

*cito:EntityMetadata*Metadata describing the Work itself,
including for example the name of the creator(s), the title, and the data and
place of creation.  (Subclass of *cito:Metadata.*)

*cito:ExperimentalProtocol*A predefined written procedural
method, designed to ensure successful replication of results by others in the
same or other laboratories, that describes the overall objectives, organization
and implementation of a scientific experiment, and specifies the experimental
design, experimental methods, reagents, instrumentation, sampling schedules,
data collection parameters, statistical analyses, image processing procedures,
safety precautions, reporting standards, etc. employed in undertaking the
experiment.  (Subclass of *cito:Specification.*)

*cito:GrantApplication*A formal written request for
financial support from a grant-giving body in support of a project, for example
an academic research project.  (See also *cito:CaseForSupport*.)

*cito:InstructionalWork*A Work created for the
purpose of education or instruction, such as a  text book, lecture,
tutorial or instruction manual.

*cito:Image*A visual representation other than
text, including all types of *cito:Moving Image* and *cito:Still Image*.

*cito:Metadata*A separate Work that provides
information describing one or more characteristics of another Work or of an
Expression of that Work.

*cito:MinimalInformationStandard*A
metadata standard specifying items to be included when creating metadata
describing a dataset of a particular type, or when creating a structured
digital abstract summarizing the main findings of an article or report in a
particular domain of interest, thereby ensuring adequate descriptive
information is recorded for subsequent resource discovery and/or interpretation
of the information described.  (Subclass of *cito:Specification.*)

*cito:Model*A mathematical, graphical or physical
representation of some physical reality, conceptual idea or theoretical
construct.

*cito:MovingImage*A series of visual representations
imparting an impression of motion when shown in succession.  Examples
include animations, movies, television programs, videos, and computational
simulations. (Subclass of *cito:Image*)

*cito:NewsItem*An item of news.

*cito:Ontology*A
formal representation of a set of concepts within a domain of knowledge, and
the relationships between those concepts.  (Subclass of*cito:Specification.*)

*cito:Opinion*  An expression of a personal or professional
opinion on an issue or topic.

*cito:Patent*A formal disclosure of a new
invention approved by a government, made to record registration of intellectual
property rights, and giving exclusive rights to the inventor or assignee to
manufacture, use, licence or sell the invention for a certain number of years.

*cito:PatentApplication*A formal disclosure of a new
invention, made in application for a Patent.

*cito:ProjectMetadata*Metadata describing the project that
led to the creation of the Work, for example the project name, project
institution and project funding information.  (Subclass of *cito:Metadata.*)

*cito:Proposition*  A proposal or proposition of a new
conceptualization, hypothesis, idea or theory.

*cito:ReferenceWork*A work  to which people refer
for authoritative factual information, such as a dictionary, encyclopaedia,
handbook or field guide, or certain types of informative web page, e.g. an
institutional, research group or project home page.

*cito:Report*  A formal factual, methodological, statistical,
technical or research report issued by an individual, group, agency, government
body or other institution.

*cito:ReportingStandard*A set
of recommendations for the minimum reporting requirements to be employed when
reporting a particular type of investigation or project, for example a
randomized clinical trial, that may involve a checklist and a flow diagram, and
that offers a standard way for authors to prepare complete and transparent
reports of their findings, and aids their critical appraisal and interpretation
of their data.  (Subclass of *cito:Specification.*)

*cito:ResearchPaper*  A report of original research findings.

*cito:Review*  A scholarly review of others’ work.

*cito:Specification*An explicit description of, or set of
requirements to be satisfied by a material, product resource, service or
standard.

*cito:StandardOperatingProcedure*Clear and detailed written
instructions of a prescribed step-by-step procedure to be routinely followed
and decisions to be made when undertaking a specific task, process or function,
to achieve consistent performance, ensure safety and/or assure data quality. 
(Commonly abbreviated "SOP".)  (Subclass of*cito:Specification.*)

*cito:StillImage*  A static visual representation, including
diagrams, drawings, graphic designs, plans, maps, photographs and prints.
(Subclass of *cito:Image.* Use where appropriate in preference to the
superclass *cito:Image*.)

*cito:Taxonomy*A
classification arranged in a hierarchical structure of classes and subclasses,
showing parent-child *isA* relationships, or *broader\_than -* *narrower\_than*
relationships.  (Subclass of *cito:Specification.*)

*cito:TechnicalStandard*An official or public specification
or requirement for a technical method, practice, process or protocol involved
in, for example, manufacturing, computation, electronic communication, or
digital media.  (Subclass of *cito:Specification.*)

*cito:Thesis*A Work consisting of formal
presentations of research outputs submitted for examination in completion of a
course of study at an institution of higher education, to fulfil the
requirements for an academic degree.  [For the alternative meaning of the
word "Thesis", namely a formulation of a concept, hypothesis, idea,
point of view or theory presented for review and/or discussion, use *cito:Proposition*.]

*cito:Workflow*A recorded sequence of connected
steps, which may be automated, specifying a reliably repeatable sequence of
operations to be undertaken when conducting a particular project, for example
an in silico investigation that extracts and processes information from a
number of bioinformatics databases.  (Subclass of *cito:Specification.*)

*cito:WorkingPaper*An unpublished paper, usually
circulated privately among a small group of peers, to provide information or
with a request for editorial improvement.

**CiTO Expression,
specifying the nature or type of the Expression of a Work, and its sub-classes**

*cito:Expression*As defined by FRBR (http://www.ifla.org/VII/s13/frbr/frbr1.htm),
an Expression is the specific form that a Work takes each time it is 'realized'
in physical or electronic form.  For CiTO, it is usually the final
published Expression of the Work, known to publishers as the 'version of
record', which is critical, since it is the references within this version of
record that are definitive, and it is the version of record itself that is
cited by others.  However, when citing Web Information Objects, it is
their Manifestations that are normally the targets of the citations.

When using CiTO, an
Expression should be assigned to one of the following **43 disjoint sub-classes**:

*cito:Abstract*A brief summary of a Work that gives
an in-depth description or analysis of a particular subject (typically a
Research Paper, Report, Review or Thesis), appearing at the beginning of the
Work and designed to act as the point-of-entry that will help the reader
quickly to obtain an overview of its contents.  (Subclass of*cito:Document*.)

*cito:BlogEntry*Information manifested in a Blog, a
type of WebPage comprising  periodic sequential entries containing
commentary, descriptions of events, or other material such as images or videos,
usually displayed in reverse-chronological order and usually maintained by an
individual. (Subclass of *cito:WebContent*.)

*cito:Book*  A
non-serial publication that is complete in one volume or a designated finite
number of volumes.  A printed Manifestation of a book is often identified
with an ISBN. (Subclass of *cito:Document*.)

*cito:BookChapter*A defined chapter of a book, usually
with a separate title or number. (Subclass of *cito:BookSection*.)

*cito:BookSection*A defined section of a book, such as
a preface, chapter or index, usually with a separate title or number.
(Subclasses of *cito:Document.*)

*cito:CaseForSupportDocument*An Expression of a Case for Support.
(Usually associated with a Grant Application Document.)  (Subclasses of*cito:Document.*)

*cito:ConferencePaper*  A Research Paper, Review, Report or other work
presented at a conference, seminar, symposium, workshop or similar event.
(Subclasses of *cito:Document.*)

*cito:ConferencePoster*A poster submitted for acceptance to
and/or presented at a conference, seminar, symposium, workshop or similar
event. (Subclasses of *cito:Document.*)

*cito:ConferenceProceedings*A publication containing the
programme and collected Conference Papers presented at a conference, seminar,
symposium, workshop or similar event, or their Abstracts*. (Subclasses of
cito:Document.)*

*cito:Database*A structured collection of logically
related records or data stored and retrieved using computer-based means.

*cito:Document*An Expression of a Work conveying a
body of information primarily in textual form.  A Document may contain one
or more Figures and Tables in addition to text.

*cito:Editorial*The
Opinions of an editor expressed in a published Periodical Issue. (Subclass of*cito:PeriodicalItem.*)

*cito:Email*A Personal Communication transmitted
over the Internet as an item of electronic mail, typically based on the Simple
Mail Transfer Protocol (SMTP).  (Subclasses of *cito:Document.*) 
 

*cito:Figure*A visual communication object
comprising one or more StillImages on a related theme.  If included within
a publication, a Figure is typically unaligned with the main body of text,
having its own descriptive textual figure legend.

*cito:GrantApplicationDocument*An Expression of a Grant Application.
(Usually associated with a Case For Support Document.) (Subclasses of*cito:Document.*)

*cito:Journal*A scholarly Periodical primarily
devoted to the publication of original Research Papers.  (Subclass of*cito:Periodical*.)

*cito:JournalArticle*A Research Paper, Discussion,
Opinion, Proposition or Review published in a scholarly
Journal.    (Subclass of *cito:PeriodicalItem*.) 
[Where the contribution is another type of journal item, e.g. an Editorial,
News Item or Letter, use the disjoint class *cito:JournalItem*.]  

*cito:JournalIssue*A particular published issue of a
Journal, one or more of which will comprise a volume of the Journal. 
(Subclass of *cito:PeriodicalIssue*.)

*cito:JournalItem*An item in scholarly Journal, for
example an Editorial, NewsItem,  Research Paper or Review. (Subclass of*cito:PeriodicalItem*.)

*cito:Letter*A Personal Communication of a
personal or professional nature between individuals and/or representatives of
corporate bodies, typically in written or printed form and usually transmitted
by the postal service or published in a Periodical.  In the latter case,
the letter is typically addressed to the Editor and comments on or discussed an
item previously published by that Periodical, or of interest to its readership.
(Subclasses of *cito:Document*.)

*cito:Magazine*A Periodical, usually devoted to a
particular topic or domain of interest, and usually published weekly or
monthly, consisting primarily of  non-peer reviewed Editorials,
journalistic News Items and more substantive Articles, Reviews, Book Reviews
and Discussions concerning current or recent events and publications, and
matters of interest to the domain served by the Magazine.  Some scientific
Journals, notably *Science* and *Nature*, also serve as science
Magazines, containing substantive news reports and articles that that describe
medical and scientific breakthroughs of vital or controversial
importance.  (Subclass of *cito:Periodical*.)

*cito:MagazineArticle*An article published in a Magazine,
that is typically not peer-reviewed.  (Subclass of *cito:PeriodicalItem*.)

*cito:MagazineIssue*A particular published  issue of
a Magazine, identified by date, and sometimes also by place (e.g.
"European edition") or language (e.g. "Spanish
edition").  (Subclass of *cito:PeriodicalIssue*.)

*cito:Manuscript*A textual Work prepared by hand, such
as a typescript or word-processed pre-publication draft of a Research Paper or
a Report, or a Work not otherwise reproduced in multiple copies.
[cito:Manuscript is not intended to describe a handwritten historical document
on paper or parchment, for which the distinction between Expression,
Manifestation and Copy becomes blurred.] [See also *cito:Preprint.*]
(Subclass of *cito:Document*.)

*cito:Newspaper*A non-peer reviewed Periodical,
usually published daily or weekly, consisting primarily of Editorials and News
Items concerning current or recent events and matters of public interest. 
(Subclass of *cito:Periodical*.)

*cito:NewspaperArticle*An article written by a journalist
and published in a Newspaper.  (Subclass of *cito:PeriodicalItem*.)

*cito:NewspaperIssue*A particular published  issue of
a Newspaper, identified by date, and sometimes also by place or time (e.g.
"Late London Edition").  (Subclass of *cito:PeriodicalIssue*.)

*cito:PatentApplicationDocument*The physical or electronic Expression
of a Patent Application.  (Subclasses of *cito:Document.*)

*cito:PatentDocument*The physical or electronic Expression
of a Patent.  (Subclasses of *cito:Document.*)

*cito:Periodical*A publication issued on a regular and
ongoing basis, comprised of separate Editorials, Articles, News Items and/or
other writings*.*

*cito:PeriodicalIssue*A particular issue of a Periodical,
identified and distinguished from other issues of the same publication by date
and/or issue number and/or volume number. (Subclass of *cito:Document*.)

*cito:PeriodicalItem*A piece of writing published in a
Periodical, typically accompanied by other items by different authors.
(Subclass of *cito:Document*.)

*cito:PersonalCommunication*Information communicated personally
from one individual to one or more another persons or organizations.

*cito:Preprint*An author’s original scholarly Work
as submitted to and/or accepted by a Journal for publication.  [For the
version of the article published by the Journal and forming the ‘version of
record’, use *cito:JournalArticle*.  See also *cito:Manuscript.*] 
(Subclass of *cito:Document*.)  

*cito:Presentation*A set of images of text and/or
pictures used when communicating ideas or research results to an audience at a
meeting, conference, symposium, seminar, lecture, workshop or other gatherings.

*cito:PressRelease*A
News Item published by an organization to provide information to journalists.
(Subclass of *cito:Document*)

*cito:ReportDocument*The embodiment of a Report, usually
in printed form. (Use when the Report is not expressed in a Book, Book Section,
Journal Item or other more specific form of Expression.)  (Subclasses of*cito:Document.*)

*cito:Software*A
computer program in source or compiled form, employing one or more Algorithms
to execute a particular task.

*cito:Spreadsheet*A computer file used for data storage
that displays a grid of rows and columns, in which each cell can contain
alphanumeric text, a numeric value, or a formula that defines how the content
of that cell is to be calculated from the content of any other cell or cells.

*cito:SupplementaryInformation*A document accompanying a published
JournalArticle, containing additional information of relevance to the article,
typically available from the publisher's web site via the JournalArticle itself.
(Subclasses of *cito:Document.*)

*cito:Table*A graphical means of presenting data
in a grid of rows and columns, within which the cells usually contain
alphanumeric text or numeric values.  If included within a publication, a
table typically appearing unaligned with the main body of text, with its own
descriptive title.

*cito:WebContent*Information prepared specifically and
primarily for manifestation in a WebPage, comprising Text, Images, Tables
and/or other Works.

*cito:WikiEntry*Information manifested in a Wiki, a
type of WebPage that enables easy editing, usually maintained collaboratively
by a project team, group or community to accumulate related information for
shared use by the group, and for publication.  (Subclass of*cito:WebContent*.)

**CiTO Manifestation,
specifying the Manifestation of an Expression  of a Work, and its
sub-classes**

*cito:Manifestation*  As defined by FRBR (http://www.ifla.org/VII/s13/frbr/frbr1.htm),
a Manifestation of an Expression of a Work defines its particular embodiment.
cito:Manifestation specifically applies to electronic (digital) as well as to
physical Manifestations of Expressions.  Examples of different
Manifestations of a single 'version of record' Expression of a scholarly Work
include an Article in a print Journal, the on-line version of that article as
an HTML Web Page, and the downloadable PDF version of the same article, which
can be seen as alternate 'containers' or 'channels' for the same information.

When
using CiTO, a Manifestation of an Expression of a Work may be left unspecified,
or may be categorized as belonging to one of  the following **5 disjoint sub-classes**:

*cito:ComputerFile*A block of digital information
created by a computer system and stored electronically on a digital memory
device in a form available for re-use, typically encoded in a particular format
by a specific software application (e.g. in PDF, plain text, image, video or
word processor file format).

*cito:DigitalMediaFile*A Computer File stored off-line on a
removable magnetic, optical, solid state or other digital storage device (e.g.
a DVD, CD-ROM, DV tape, or USB flash drive) used to save, transport, distribute
and play back such files. (Subclass of *cito:ComputerFile*.)

*cito:OnlineFile*  A Computer File stored on a computer attached
to the Internet, available for viewing in a Web browser or for electronic
transfer ('uploading' or 'downloading') to a second computer across the
Internet. (Subclass of *cito:ComputerFile*.)

*cito:PrintObject*  A Manifestation of information in physical
printed form, typically on paper.

*cito:WebPage*  A Manifestation of information on the World
Wide Web, usually structured in HTML or XHTML format, identified by a Uniform
Resource Identifier (URI), and made accessible to a user by means of the
Hypertext Transport Protocol in a Web browser window.  Special software
may enable a WebPage to be or to include a Blog or a Wiki.  Several
interlinked WebPages hosted together on a Web server and accessed through a
single domain name or IP address constitute a Web site.

**CiTO Data Property
relevant to the Publication Status and Peer Review Status of the Expression of
a Work**

*cito:peerReviewed*  A Boolean data property having the value 'True'
if the cited Work has been peer reviewed, or 'False' if the cited Work has not
been peer reviewed.  [When using CiTO, the truth value of *cito:peerReviewed*
may be left unspecified.] (Domain: *cito:Expression*.)

*cito:unpublished*A Boolean data property having the
value 'True' if the cited Work has not been published, or 'False' if the cited
Work has been published.  [When using CiTO, the truth value of *cito:Unpublished*
may be left unspecified.] (Domain: *cito:Expression*.)

**CiTO Classes and
Properties relevant to Citation Frequencies**

**Classes:**

*cito:GlobalCitationCount*The number of times a Work has been
cited globally, as determined from a particular bibliographic information
source on a particular date.

*cito:InTextCitationCount*  The number of times a cited Work has been cited
within the text of a citing Work.

**Object Properties:**

*cito:globalCitationFrequency*The property linking an Expression of
a Work to its Global Citation Count*.*

*cito:globalCountSource*The URI of the bibliographic
information source of information providing the Global Citation Count of a
Work.

*cito:inTextCitationFrequency*The property linking an Expression of
a cited Work to its In-Text Citation Count within the citing Work.

*cito:inTextCitationTarget*The URI of the cited *Work* for
which the In-Text Citation Count is relevant*.*

**Data Properties:**

*cito:globalCountDate*The date on which the Global Citation
Count of the Work was recorded from a named bibliographic information source.

*cito:globalCountValue*  An integer defining the value of the Global
Citation Count of the Work recorded from a named bibliographic information
source on a particular date.

*cito:inTextCountValue*  An integer defining the value of the In-Text
Citation Count of the cited Work within the citing Work.

**CiTO Data Property
relevant to Work, Expression and Manifestation**

*cito:isRealizationOf*As defined by FRBR (http://www.ifla.org/VII/s13/frbr/frbr1.htm),
the relationship between an Expression and the Work that is being expressed.
(Domain: *cito:Expression*, Range: *cito:Work*.  Inverse
property: *cito:isRealizedThrough.*)

*cito:isEmbodimentOf*As defined by FRBR (http://www.ifla.org/VII/s13/frbr/frbr1.htm),
the relationship between a Manifestation and the Expression being manifested.
(Domain: *cito:Manifestation*, Range: *cito:Expression.* Inverse
property: *cito:isEmbodiedIn.*)

*cito:isEmbodiedIn*  As defined by FRBR (http://www.ifla.org/VII/s13/frbr/frbr1.htm),
the relationship between an Expression of a Work and its Manifestation.
(Domain: *cito:Expression*, Range: *cito:Manifestation.* Inverse
property: *cito:isEmbodimentOf.*)

*cito:isRealizedThrough*As defined by FRBR (http://www.ifla.org/VII/s13/frbr/frbr1.htm),
the relationship between a Work and its Expression. (Domain: *cito:Work*,
Range: *cito:Expression*.  Inverse property: *cito:isRealizationOf.*)

**Changes between version
1.3 and version 1.6 of CiTO**

**3 New FRBR classes**

*frbr:Work*

*frbr:Expression*

*frbr:Manifestation*

**9 New CiTO Object
Properties**

*cito:citesAsAuthority*

*cito:citesAsMetadataDocument*

*cito:citesAsSourceDocument*

*cito:qualifies*

*cito:supports*

*cito:isRealizedThrough             (Domain:
cito:Work, Range: cito:Expression. 
Inverse property: cito:isRealizationOf.)*

*cito:isRealizationOf                    (Domain:
cito:Expression, Range: cito:Work. 
Inverse property: cito:isRealizedThrough.)*

*cito:isEmbodiedIn                       (Domain:
cito:Expression, Range: cito:Manifestation. 
Inverse property: cito:isEmbodimentOf.)*

*cito:isEmbodimentOf                  (Domain:
cito:Manifestation, Range: cito:Expression.  Inverse property:
cito:isEmbodiedIn.)*

**1 New CiTO Data Property**

*cito:unpublished* *(Domain:* *cito:Expression**.)*

**Change to CiTO Object
Properties and Data Properties**

Domain
and range restrictions removed on *cito:cite*
and its sub-properties, and on *cito:isCitedBy*.

Domain
restrictions removed on *cito:globalCitationFrequency*
and *cito:inTextCitationFrequency*.

Range restriction removed on
*cito:inTextCitationTarget*.

**20 New Subclasses of *CiTO:Work***

*cito:Algorithm*

*cito:AuthorityFile* (Subclass of *cito:Specification*)

*cito:BibliographicMetadata* (Subclass of *cito:Metadata*)

*cito:Biography*

*cito:CaseForSupport*

*cito:CitationMetadata*(Subclass of *cito:Metadata*)

*cito:ClinicalCaseReport* (Subclass of *cito:Report*)

*cito:ClinicalTrialDesign*(Subclass of *cito:Specification*)

*cito:ClinicalTrialReport* (Subclass of *cito:Report*)

*cito:EntityMetadata*(Subclass of *cito:Metadata*)

*cito:InstructionalWork*

*cito:
Metadata*

*cito:MinimalInformationStandard* (Subclass of *cito:Specification*)

*cito:PatentApplication*

*cito:ProjectMetadata*(Subclass of *cito:Metadata*)

*cito:ReportingStandard* (Subclass of *cito:Specification*)

*cito:StandardOperatingProcedure*(Subclass of *cito:Specification*)

*cito:TechnicalStandard*            (Subclass of *cito:Specification**)*

*cito:Thesis*                                                    (Originally mis-classified
as a Subclass of *cito:Expression*)

*cito:Workflow*(Subclass of *cito:Specification*)

**3 Renamed Subclasses of *CiTO:Work***

*cito:Catalog*(Formerly *cito:Catalogue*)

*cito:ExperimentalProtocol*(Formerly *cito:Protocol*)

*cito:Proposition*(Formerly *cito:Explanation*)    

**5 Deprecated Subclass of *CiTO:Work***

*cito:Discussion*  (Deleted)

*cito:Editorial*                                 
                        (Originally mis-classified, now placed as a Subclass of
*cito:Expression*)

*cito:Message*                                
           (Deleted)

*cito:Software*
                               
                         (Originally mis-classified, now placed as a Subclass
of *cito:Expression*)

*cito:ScholarlyText*                   
              (Deleted)

**26 New
Subclasses of** ***CiTO:Expression***

*cito:Abstract* (Subclass of *cito:Document*)

*cito:CaseForSupportDocument*(Subclass of *cito:Document*)

*cito:ConferenceProceedings*(Subclass of *cito:Document*)

*cito:Document*

*cito:Editorial*  (Previously mis-classified as a subclass
of *cito:Work*)

*cito:GrantApplicationDocument* (Subclass of *cito:Document*)

*cito:Journal*  (Subclass of *cito:Periodical*)

*cito:JournalIssue* (Subclass of *cito:PeriodicalIssue*)

*cito:Letter*  (Subclass of *cito:Document*)

*cito:Magazine*  (Subclass of *cito:Periodical*)

*cito:MagazineArticle*  (Subclass of *cito:PeriodicalItem*)

cito:MagazineIssue            
              
     (Subclass of cito:PeriodicalIssue)

*cito:Manuscript*  (Subclass of *cito:Document*)

*cito:Newspaper*  (Subclass of *cito:Periodical*)

*cito:NewspaperArticle*  (Subclass of *cito:PeriodicalItem*)

*cito:NewspaperIssue*  (Subclass of cito:PeriodicalIssue)

*cito:PatentApplicationDocument* (Subclass of *cito:Document*.)

*cito:Periodical*

*cito:PeriodicalIssue*  (Subclass of *cito:Document*)

*cito:PeriodicalItem*  (Subclass of *cito:Document*)

*cito:PersonalCommunication*

*cito:PressRelease*  (Subclass of *cito:Document*)

*cito:Software*  (Previously mis-classified as a
Subclass of *cito:Work*)

*cito:SupplementaryInformation*  (Subclasses of *cito:Document*)

*cito:WebContent*

*cito:WikiEntry*  (Subclass of *cito:WebContent*)

**1 Renamed Subclass of *CiTO:Expression***

*cito:BlogEntry* (Formerly *cito:Blog*)
(Subclass of *cito:WebContent*)

**2 Deprecated Subclass of *CiTO:Expression***

*cito:TextFile*                        (Deleted)

*cito:Thesis*(Previously mis-classified, now placed as
a Subclass of *cito:Work*)

**1 New Subclasses of *CiTO:Manifestation***

*cito:ComputerFile*

**3
Renamed Subclasses of *CiTO:Manifestation***

*cito:DigitalMediaFile*(Subclass of *cito:ComputerFile*)(Formerly *cito:DigitalMediaObject*)

*cito:OnlineFile*(Subclass of *cito:ComputerFile*)(Formerly *cito:OnlineDocument*)

*cito:PrintObject*(Formerly *cito:PrintDocument*)

 

Throughout the ontology,
several class and property definitions were altered to clarify their meaning,
without changing their sense.

**Summary of changes between
published CiTO versions**

|  |  |  |  |  |
| --- | --- | --- | --- | --- |
| **Item** | **CiTO v1.3** | **CiTO v1.4** | **CiTO v1.5** | **CiTO v1.6** |
| Object properties | 22 | 25 | 31 | 31 |
| Data properties | 4 | 4 | 5 | 5 |
| Classes | 55 | 72 | 100 | 98 |
| Sub-classes of *cito:Work* | 26 | 30 | 41 | 41 |
| Sub-classes of *cito:Expression* | 19 | 29 | 43 | 43 |
| Sub-classes of *cito:Manifestation* | 4 | 6 | 9 | 5 |

**Acknowledgements**

The
contributions to this work of Katie Portwin, who participated in the
development of the initial CiTO prototype, and of Alistair Miles and Graham
Klyne, for helpful discussions on ontology structure, guidance in RDF modelling
and syntax, and help in ontology coding and publication, are gratefully
acknowledged. 

The
development of CiTO forms part of the work of the Ontogenesis Network, a
network of excellence to foster the creation, ontogeny and evolution of
biological, bioinformatics and medical ontologies, supported by EPSRC grant
EP/E021352/1.

/end
